# Patients’ Perspectives on Pharmaceutical Care Interventions in a Secondary-Care Hospital in Nigeria: A Cross-Sectional Survey

**DOI:** 10.7759/cureus.103859

**Published:** 2026-02-18

**Authors:** Omolayo T Umaru, Taiwo O Aremu

**Affiliations:** 1 Pharmaceutical Care and Health Systems, College of Pharmacy, University of Minnesota, Minneapolis, USA; 2 Environmental Health Sciences, School of Public Health, University of Minnesota, Minneapolis, USA; 3 Pediatrics, Medical School, University of Minnesota, Minneapolis, USA; 4 Clinical and Social Pharmaceutical Sciences, College of Pharmacy, Touro University California, Vallejo, USA

**Keywords:** chronic disease management, hospital pharmacy, low-and-middle-income countries, medication counseling, nigeria, patient education, patient perspectives, patient satisfaction, pharmaceutical care

## Abstract

Background

Pharmaceutical care aims to optimize medication use and improve patient outcomes through pharmacist-led interventions. Evidence on patients' perspectives on such services remains limited in many hospital settings in low- and middle-income countries (LMICs), such as Nigeria.

Objective

This study aims to assess patients' satisfaction with pharmacists' services, the perceived impact of pharmacist-led interventions, and patient-reported gaps in pharmaceutical care service delivery in a secondary-care hospital in Ibadan, Nigeria.

Methods

A descriptive cross-sectional survey using convenience sampling was conducted among adult patients with chronic conditions attending specialist outpatient clinics and obtaining medicines from the hospital pharmacy (June-July 2017). A structured, paper-based questionnaire was developed and used to assess satisfaction with pharmacy services and the perceived impact of pharmacist interventions. Descriptive statistics were calculated, and chi-square tests explored associations between patient characteristics and overall satisfaction with pharmaceutical care activities (p < 0.05).

Results

Of the 110 questionnaires distributed, 103 (93.6%) were complete and analyzed. Overall satisfaction with pharmacist services was reported by 86 (83.5%) respondents. Most respondents (89, 86.4%) reported that pharmacists demonstrated sufficient knowledge, and 87 (84.5%) of them reported that pharmacists helped to improve their understanding of their medical condition. Reported impacts of pharmacist interventions included support for self-management (80, 77.7%), guidance on dietary adjustments (81, 78.6%), and lifestyle modifications (69, 67.0%). Counselling on foods to avoid and medicines to avoid was reported by 84 respondents (81.6%) for each of these interventions. Fewer respondents reported receiving instruction on device use or medication administration techniques (24, 23.3%), while approximately half (52, 50.5%) indicated receiving education on clinical goals, most commonly blood pressure targets (41, 39.8%). Pharmacist counseling was perceived to prevent medication discontinuation by 44 (42.7%) respondents, and educational level (p = 0.015) and monthly income (p = 0.035) were associated with overall satisfaction.

Conclusion

Patients reported high satisfaction and substantial perceived educational and supportive benefits from pharmacist services; however, gaps were noted in device or medication-administration training, and education on desired clinical goals. Strengthening structured counseling and practical demonstration components may further improve pharmaceutical care delivery.

## Introduction

Pharmaceutical care is a patient-centered practice model in which pharmacists accept responsibility for optimizing medication therapy and achieving outcomes that improve patients' quality of life. In contrast to a product-focused dispensing approach, pharmaceutical care emphasizes establishing a professional relationship with the patient, collecting and evaluating patient-specific information, identifying and resolving medication-related problems, and collaborating with other health professionals to achieve agreed therapeutic goals [1].

The expansion of pharmacists' roles from medicines supply to direct patient care has been driven by the growing complexity of pharmacotherapy and the rising burden of chronic diseases [2,3]. Patients managing long-term conditions often use multiple medicines, require ongoing monitoring, and may experience challenges such as side effects, complex regimens, and uncertainty about how to integrate medicines with daily routines [4]. In this context, pharmacist-led counseling, follow-up, and problem-solving can complement physician care and strengthen continuity across repeated outpatient encounters [2,5].

Patient satisfaction is a key humanistic outcome in pharmaceutical care delivery and is frequently used as an indicator of perceived service quality [6]. Satisfaction reflects the extent to which services meet patient needs and expectations, including access to information, respect, communication, and the perceived helpfulness of counseling. Although satisfaction is an important outcome in its own right, it can also be associated with care experiences and health outcomes, highlighting the importance of measuring and improving patient-facing services [7].

In outpatient settings, the pharmacy is a critical point of contact for patients with chronic diseases such as hypertension, diabetes, asthma, and mental health disorders. For many patients, pharmacy encounters represent the most frequent interaction with a health professional between clinic visits. Pharmacists can therefore play an important role in reinforcing disease and medicine understanding, supporting self-management, and addressing adherence barriers. Effective counseling is also central to these activities, as inadequate counseling can lead to improper medication administration, early treatment discontinuation, and medication errors that compromise therapeutic outcomes [8]. Furthermore, evidence from chronic disease literature suggests that education and support can strengthen patients' confidence and capacity to manage their conditions and may contribute to improved adherence and self-management behaviors [9,10].

In Nigeria, pharmaceutical care is evolving from a traditional dispensing role toward more comprehensive patient care. Studies in Nigerian community pharmacy settings indicate that patients value pharmacist services and report high satisfaction [11], and hospital-based studies in Northern Nigeria have similarly documented generally high satisfaction with services [12]. However, there remains limited documentation of patients' perspectives on pharmaceutical care interventions delivered within secondary-care outpatient hospital pharmacies, where workflow constraints and limited space may shape the type and depth of counseling provided.

Importantly, satisfaction scores should be interpreted within the local context. Prior work suggests that in resource-limited settings, patients may report high satisfaction even when service gaps exist, potentially reflecting low expectations, cultural norms, or limited alternative options [13]. Complementing overall satisfaction measures with domain-specific questions on counseling content, understanding, self-management support, and medication safety can therefore help identify actionable priorities for quality improvement.

Medication safety is a core component of pharmaceutical care. Counseling on correct medicine use, potential interactions, and adverse effects can reduce avoidable harm and support early recognition of problems requiring clinical review [14]. In settings where patients may obtain medicines from multiple sources or use complementary therapies, explicit counseling about medicine combinations and what to avoid may be particularly important.

Rationale for the study

Despite growing recognition of the importance of pharmaceutical care, there is limited documentation of patient perspectives on these services in Nigerian hospital settings. Understanding how patients perceive and experience pharmaceutical care interventions is important for evaluating the effectiveness of current pharmaceutical care delivery, identifying gaps in service provision from the patient perspective, informing strategies to enhance patient-centered care, and demonstrating the value of pharmaceutical care to healthcare administrators and policymakers. This study sought to address this knowledge gap by examining patients’ perspectives on pharmaceutical care interventions provided at a secondary-care hospital in Nigeria.

Study objectives

The objectives of this study were to assess patients' satisfaction with pharmaceutical care services provided at the hospital pharmacy, evaluate the perceived impact of pharmacist interventions on patients' understanding of their conditions and medications, identify patient-reported gaps in pharmaceutical care service delivery, and examine relationships between patient characteristics and satisfaction with pharmacy services. These objectives informed questionnaire development and guided the analysis.

## Materials and methods

Study setting

The study was conducted at the University Health Services Department (Jaja Clinic), a 50-bed secondary-care hospital located within the University of Ibadan, Nigeria. The facility was upgraded from primary- to secondary-care status in 2016 and provides outpatient specialist services, including clinics for hypertension, diabetes, asthma, psychiatry, and obstetrics and gynecology. The hospital pharmacy provides medication dispensing, patient counseling, medication therapy management, and drug information services to outpatients attending specialist clinics. The pharmacy also distributes medications and medical supplies to the wards for inpatient care.

Study design and participants

A descriptive cross-sectional survey was conducted over a five-week period between June and July 2017. The study population comprised adult patients with chronic conditions attending hypertension, diabetes, asthma, or psychiatry clinics and obtaining their medicines from the hospital pharmacy. Eligible participants were staff of the University of Ibadan or their dependents who provided informed consent. Type 1 diabetes patients were excluded due to distinct disease management needs and lower prevalence in the study population. Students were excluded as they represent a transient population with different healthcare utilization patterns. Patients not obtaining medicines from the hospital pharmacy were excluded as the study focused on pharmaceutical care delivered by the hospital pharmacy.

Sampling and sample size

Convenience sampling was used to recruit participants during clinic visits within the study period. A total of 110 questionnaires were distributed; 103 complete questionnaires were included in the analysis.

Data collection instrument

A structured, paper-based questionnaire was developed to assess patient satisfaction with pharmacy services and the perceived impact of pharmacist interventions. Item construction was informed by the publicly available Hospital Consumer Assessment of Healthcare Providers and Systems (HCAHPS) survey materials [15]. Relevant items were adapted to the outpatient pharmacy context. The HCAHPS survey questions are in the public domain and may be used without a licensing fee; accordingly, copyrighted Care Transitions Measure items and proprietary supplemental questions were not included. Face validity was assessed by two practicing pharmacists, and the questionnaire was pretested with two patients prior to use. Most items used five-point Likert-type responses.

Data collection procedure

Patients attending outpatient clinics were screened for eligibility based on the inclusion/exclusion criteria. Consenting eligible patients were provided with paper questionnaires. Two research assistants supported questionnaire distribution and collection. Most questionnaires were completed immediately and returned on-site; those not completed immediately were collected at subsequent clinic visits. Of 110 questionnaires distributed, 103 were complete and included in the analysis; seven incomplete questionnaires were excluded.

Data analysis

Data were entered into a spreadsheet and imported into SPSS version 18.0 (Released 2009; SPSS Inc., Chicago, IL, USA) for analysis. Descriptive statistics (frequencies and percentages) summarized responses. For analysis, Likert-scale satisfaction responses (strongly agree, agree, not sure, disagree, strongly disagree) were collapsed into three categories: dissatisfied (combining disagree and strongly disagree), not sure, and satisfied (combining agree and strongly agree). Chi-square tests explored associations between satisfaction and selected patient characteristics; p < 0.05 was considered statistically significant. Educational level, monthly income, and years of hospital attendance were examined as they represent key socioeconomic and service familiarity factors that were feasible to measure within the study design; other variables may also influence satisfaction but were not tested in this exploratory analysis.

Ethical consideration

Participants were informed about the study's purpose and confidentiality, and no personal identifiers were collected. Institutional approval was obtained from the Director, University of Ibadan Health Services, and ethical approval was obtained from the Oyo State Ministry of Health Ethical Review Committee (Ref No. AD 13/479/563).

## Results

Of the 110 questionnaires distributed, 103 were complete and analyzed (response rate was 93.6%).

Participant characteristics

Demographic characteristics of participants are summarized in Table 1. Of the 103 respondents, 55 (53.4%) were females. The predominant age groups were 40-49 years (32, 31.1%), 50-59 years (23, 22.3%), and 60-69 years (21, 20.4%). Most were senior staff (37, 35.9%) or pensioners (22, 21.4%). Monthly income was less than ₦20,000 for 35 (34.0%) respondents and between ₦100,000 and ₦200,000 for 21 (20.4%) of them. Educational levels ranged from primary school (22, 21.4%) to master's degrees (27, 26.2%). Over half (58, 56.3%) had attended the hospital for more than 10 years. Most attended the hypertension clinic (62, 60.2%) or diabetes clinic (20, 19.4%). Seventy (68.0%) were enrolled in the National Health Insurance Scheme.

**Table 1:** Demographic characteristics of respondents (N = 103)

Characteristics	Categories	n	%
Gender	Male	48	46.6
	Female	55	53.4
Age (years)	20-29	9	8.7
	30-39	15	14.6
	40-49	32	31.1
	50-59	23	22.3
	60-69	21	20.4
	70 and above	3	2.9
Employment status	Junior staff	9	8.7
	Senior staff	37	35.9
	Business owner	8	7.8
	Private sector	5	4.9
	Charity/Religious organization	4	3.9
	Pensioner	22	21.4
	Unemployed	12	11.7
	Dependent	5	4.9
	Other	1	1.0
Monthly income (NG ₦)	<20,000	35	34.0
	20,000 to <50,000	17	16.5
	50,000 to <100,000	16	15.5
	100,000 to <200,000	21	20.4
	>=200,000	14	13.6
Educational level	Primary school leaving certificate	22	21.4
	Senior secondary certificate (SSCE)	11	10.7
	National certificate of education (NCE)	6	5.8
	Ordinary national diploma (OND)	9	8.7
	Higher national diploma (HND)	4	3.9
	Bachelor's degree	14	13.6
	Master's degree	27	26.2
	MD/PharmD	4	3.9
	PhD	6	5.8
Years attending hospital	<1 year	15	14.6
	1-5 years	25	24.3
	6-10 years	5	4.9
	11-15 years	10	9.7
	16-20 years	21	20.4
	>20 years	27	26.2
Specialist clinic attended	Diabetes clinic	20	19.4
	Asthma clinic	3	2.9
	Hypertension clinic	62	60.2
	Psychiatry clinic	2	1.9
	Other/pharmacy	16	15.5
National Health Insurance Scheme (NHIS) enrollment	Yes	70	68.0
	No	33	32.0
Pays consultation fee	Yes	5	4.9
	No	86	83.5
	Sometimes	12	11.7

Satisfaction with pharmacy services

Table 2 presents satisfaction with pharmacist services. Overall satisfaction was 83.5% (86 respondents). Satisfaction with pharmacy location was 82.6% (85), while satisfaction with drug quality was 84.5% (87), and drug cost was 75.7% (78).

**Table 2:** Satisfaction with attributes of pharmacy services (N = 103) *Total satisfied = Satisfied + Very satisfied Percentages are based on N=103; 16 respondents did not rate drug cost.

Service attribute	Very dissatisfied, n (%)	Dissatisfied, n (%)	Not sure, n (%)	Satisfied, n (%)	Very satisfied, n (%)	Total satisfied*, n (%)
Pharmacy location	8 (7.8)	4 (3.9)	6 (5.8)	63 (61.2)	22 (21.4)	85 (82.6)
Quality of drugs	4 (3.9)	8 (7.8)	4 (3.9)	62 (60.2)	25 (24.3)	87 (84.5)
Cost of drugs	0 (0.0)	9 (8.7)	0 (0.0)	75 (72.8)	3 (2.9)	78 (75.7)
Overall satisfaction	6 (5.8)	3 (2.9)	8 (7.8)	48 (46.6)	38 (36.9)	86 (83.5)

Perceived impact of pharmacist interventions

Table 3 presents patient-reported impact across multiple domains. For knowledge and understanding, 89 (86.4%) of the patients reported that pharmacists demonstrated sufficient knowledge, and 87 (84.5%) reported improved understanding of their condition. Self-management support was reported by 80 (77.7%).

**Table 3:** Patient-reported impact of pharmaceutical care interventions (N = 103) *Positive response represents agree/strongly agree, OR often/very often; **negative responses represent disagree/strongly disagree, OR rarely/never; † not applicable: specific goals were recorded as follow‑up "select all that apply" items; negative and not sure responses were not assessed for these rows

Domain	Item	Positive response*, n (%)	Negative response**, n (%)	Not sure, n (%)
Knowledge and understanding	Pharmacist demonstrates sufficient knowledge	89 (86.4)	9 (8.7)	5 (4.9)
	Helped better understanding of my condition	87 (84.5)	8 (7.8)	8 (7.8)
Self-management support	Supported self-management efforts	80 (77.7)	10 (9.7)	13 (12.6)
Nonpharmacological interventions	Influenced dietary adjustments	81 (78.6)	13 (12.6)	9 (8.7)
	Informed lifestyle modifications	69 (67.0)	15 (14.6)	19 (18.4)
Prevention of drug interactions/safety	Helped prevent worsening of my condition	83 (80.5)	9 (8.7)	11 (10.7)
	Counseled on foods to avoid	84 (81.6)	10 (9.7)	9 (8.7)
	Counseled on medicines to avoid	84 (81.6)	9 (8.7)	10 (9.7)
Device/medication administration training	Was taught a device or medication-administration technique	24 (23.3)	79 (76.7)	0 (0.0)
Clinical goal education	Received education on any clinical goal	52 (50.5)	51 (49.5)	0 (0.0)
	Blood pressure goal	41 (39.8)	Not applicable†	
	Blood glucose goal	9 (8.7)	Not applicable†	
	Body mass index goal	2 (1.9)	Not applicable†	
Medication adherence support	Pharmacist counseling prevented medication discontinuation	44 (42.7)	39 (37.9)	20 (19.4)
Adverse drug reaction (ADR) support	Helped me report or resolve an ADR	22 (21.4)	50 (48.5)	31 (30.1)
Communication	Often shared information not mentioned to my physician	14 (13.6)	69 (67.0)	20 (19.4)

Regarding nonpharmacological interventions, 81 (78.6%) reported dietary adjustments and 69 (67.0%) reported lifestyle modifications. With regard to medication safety, 83 (80.5%) reported that pharmacists helped to prevent worsening of their conditions, while counselling on foods to avoid and medicines to avoid was reported by 84 respondents (81.6%) for each intervention.

On the other hand, only 24 (23.3%) reported receiving device or medication‑administration training. Education on clinical goals was reported by 52 (50.5%), including blood pressure goals by 41 (39.8%), blood glucose goals by nine (8.7%), and body mass index goals by two (1.9%).

For adherence support, 44 (42.7%) agreed that pharmacist counseling prevented medication discontinuation, while 39 (37.9%) disagreed and 20 (19.4%) were unsure. Adverse drug reaction support was reported by 22 (21.4%), with 50 (48.5%) reporting no support and 31 (30.1%) unsure. Fourteen respondents (13.6%) often shared information with pharmacists not mentioned to physicians, while 69 (67.0%) rarely or never did so.

Factors associated with patients' satisfaction with pharmaceutical care

Table 4 presents chi-square analyses examining relationships between patient characteristics and satisfaction with pharmacists’ services. Educational level (χ² = 29.486, df = 16, p = 0.015) and monthly income (χ² = 16.438, df = 8, p = 0.035) showed statistically significant relationships with satisfaction. Years of hospital attendance showed no significant relationship (χ² = 6.824, df = 10, p = 0.792).

**Table 4:** Factors associated with overall satisfaction (N = 103) χ² values are Pearson chi-square statistics; degrees of freedom (df) are shown in parentheses. Statistical significance was defined as p < 0.05

Characteristic	χ² (df)	p-value	Interpretation
Educational level	29.486 (16)	0.015	Lower education associated with higher satisfaction
Monthly income	16.438 (8)	0.035	Lower income associated with higher satisfaction
Years attending hospital	6.824 (10)	0.792	No significant association

## Discussion

Overall satisfaction with pharmacist services was high, with respondents reporting substantial perceived benefits from pharmacist-delivered education and counseling. These findings reinforce the importance of pharmacists' educational and supportive roles beyond routine dispensing [1,6]. Key areas of perceived benefit included improved understanding of medical conditions, self-management support, and prevention of harmful food-drug and drug-drug interactions.

High satisfaction ratings should be interpreted alongside the context in which patient expectations and service norms influence satisfaction scores. Prior studies have noted that in resource-limited settings, patients may report high satisfaction even when service constraints exist, potentially reflecting modest expectations or cultural influences on ratings [12,13]. Nevertheless, the consistently high positive responses across multiple educational and safety-related items suggest that pharmacist interactions were meaningful to many respondents.

Respondents perceived pharmacists to have sufficient knowledge and reported that pharmacists helped them better understand their conditions. This is consistent with evidence that pharmacist counseling and education can enhance patient knowledge, which is foundational to effective self-management in chronic diseases [9,10]. Similarly, patient activation and confidence in self-management have been associated with better health behaviors and care experiences [10].

Frequent counseling on foods and medicines to avoid, along with perceived prevention of condition worsening, suggests that pharmacists are fulfilling important medication-safety functions. Education on drug-food and drug-drug interactions is known to be an important medication-safety function, as such interactions contribute significantly to adverse events in outpatient care [14].

Two important gaps were, however, identified: First, a few respondents reported receiving device or medication-administration training. Skills training is particularly important, for instance, correct inhaler technique, insulin injection, or home monitoring procedures; incorrect administration is common and compromises disease control [16-18]. Second, only half of the respondents reported receiving education on clinical goals such as blood pressure and blood glucose targets. Knowledge of these goals has been known to support self-monitoring and timely care-seeking and has been associated with improved control in chronic diseases [19,20]. These findings suggest opportunities to strengthen practical skills demonstration and goal-setting education within pharmaceutical care encounters.

Reports about adherence support were mixed. This suggests that even though pharmacist counseling may influence adherence for a substantial subgroup, adherence is multifactorial, being shaped by beliefs, costs, regimen complexity, and health system factors [21,22]. Other multicomponent strategies proposed to strengthen adherence include patient- and provider-focused education, improved communication (including reminders), community-based support, and reducing out-of-pocket barriers [23].

Regarding adverse drug reactions, fewer respondents recalled receiving pharmacovigilance support. This indicates an opportunity to strengthen routine pharmacovigilance by pharmacists, as well as patient education on how to recognize and report potential adverse effects.

A few respondents reported sharing information with pharmacists that they had not shared with physicians. This not only suggests limited opportunities for in-depth consultation within routine dispensing workflows but also highlights a potential missed opportunity for pharmacists to elicit medication experiences, adherence challenges, and concerns that may not be raised elsewhere.

Lower educational status and lower income were both associated with higher satisfaction, which is consistent with published studies, suggesting that patients with higher socioeconomic status may have higher expectations regarding the scope and depth of pharmaceutical care services [24,25]. These findings showcase the importance of tailoring communication and counseling approaches to diverse patient needs and expectations.

Strengths and limitations

This study provides patient-reported evidence on pharmaceutical care in a Nigerian secondary-care outpatient setting, addressing a gap in the literature. The high response rate (93.6%) and use of a structured questionnaire covering multiple domains of pharmaceutical care are additional strengths. However, the convenience sampling approach and the single-site patient population may limit generalizability. Potential biases include selection bias from convenience sampling and social desirability bias in self-reported satisfaction measures. The study relied on self-reported perceptions and did not assess objective clinical outcomes. The questionnaire was adapted and face-validated, but was not formally psychometrically validated in this study. Finally, some items had missing data (e.g., cost of drugs).

Implications for practice

Interventions to strengthen pharmaceutical care in similar settings may include: structured counseling checklists, practical demonstrations, and integration of education on clinical goals and ADR-reporting support into pharmacy visits/encounters. Such enhancements would improve the consistency and comprehensiveness of pharmacist-delivered care.

## Conclusions

Patients in this Nigerian secondary-care hospital reported high satisfaction with pharmaceutical care services and described meaningful educational and supportive benefits from pharmacist interactions. Respondents commonly appreciated improved understanding of their medical conditions and medicines, support for self-management, and guidance on diet, lifestyle modification, and avoidance of potentially harmful food-drug or drug-drug interactions. However, important gaps remained in practical skills training (e.g., device or medication administration technique) and education on individualized clinical goals. The observed associations between overall satisfaction and socioeconomic variables (education and income) suggest that expectations and information needs may differ across patient groups. Improving the completeness and consistency of pharmaceutical care in similar settings will likely require both professional and system-level actions. These may include structured counseling checklists, routine practical demonstrations for devices/administration techniques, integration of education on clinical goals into dispensing encounters, and strengthened pharmacovigilance support to help patients recognize and report suspected adverse drug reactions. Future multicenter studies should combine validated patient-reported measures with more robust qualitative enquiry and, where feasible, objective clinical or adherence outcomes to determine whether service enhancements would translate into better long-term chronic disease management.

## References

[1] Hepler CD, Strand LM (1990). Opportunities and responsibilities in pharmaceutical care. Am J Hosp Pharm.

[2] (2026). TRS 961-annex 8: joint FIP/WHO guidelines on good pharmacy practice: standards for quality of pharmacy services. https://www.who.int/publications/m/item/annex-8-trs-961.

[3] (2026). Noncommunicable diseases. https://www.who.int/news-room/fact-sheets/detail/noncommunicable-diseases.

[4] Nicholson K, Liu W, Fitzpatrick D (2024). Prevalence of multimorbidity and polypharmacy among adults and older adults: a systematic review. Lancet Healthy Longev.

[5] Rahayu SA, Widianto S, Defi IR, Abdulah R (2021). Role of pharmacists in the interprofessional care team for patients with chronic diseases. J Multidiscip Healthc.

[6] Schommer JC, Kucukarslan SN (1997). Measuring patient satisfaction with pharmaceutical services. Am J Health Syst Pharm.

[7] Kennedy GD, Tevis SE, Kent KC (2014). Is there a relationship between patient satisfaction and favorable outcomes?. Ann Surg.

[8] Aduke EI, Ajanaku OO, Umaru OT (2023). Assessment of community pharmacists’ knowledge and counselling practices on oral contraceptives use. J Contemp Stud Epidemiol Public Health.

[9] Kvarnström K, Westerholm A, Airaksinen M, Liira H (2021). Factors contributing to medication adherence in patients with a chronic condition: a scoping review of qualitative research. Pharmaceutics.

[10] Hibbard JH, Greene J (2013). What the evidence shows about patient activation: better health outcomes and care experiences; fewer data on costs. Health Aff (Millwood).

[11] Oparah AC, Kikanme LC (2006). Consumer satisfaction with community pharmacies in Warri, Nigeria. Res Social Adm Pharm.

[12] Iliyasu Z, Abubakar IS, Abubakar S, Lawan UM, Gajida AU (2010). Patients' satisfaction with services obtained from Aminu Kano Teaching Hospital, Northern Nigeria. Niger J Clin Pract.

[13] Papanikolaou V, Ntani S (2008). Addressing the paradoxes of satisfaction with hospital care. Int J Health Care Qual Assur.

[14] Cai R, Liu M, Hu Y (2017). Identification of adverse drug-drug interactions through causal association rule discovery from spontaneous adverse event reports. Artif Intell Med.

[15] SurveyMonkey SurveyMonkey (2017). CAHPS® and HCAHPS® surveys for healthcare quality improvement. https://www.surveymonkey.com/learn/healthcare/cahps-survey/.

[16] Rootmensen GN, van Keimpema AR, Jansen HM, de Haan RJ (2010). Predictors of incorrect inhalation technique in patients with asthma or COPD: a study using a validated videotaped scoring method. J Aerosol Med Pulm Drug Deliv.

[17] Frid AH, Hirsch LJ, Menchior AR, Morel DR, Strauss KW (2016). Worldwide injection technique questionnaire study: population parameters and injection practices. Mayo Clin Proc.

[18] Frid AH, Hirsch LJ, Menchior AR, Morel DR, Strauss KW (2016). Worldwide injection technique questionnaire study: injecting complications and the role of the professional. Mayo Clin Proc.

[19] DeVore AD, Sorrentino M, Arnsdorf MF, Ward RP, Bakris GL, Blankstein R (2010). Predictors of hypertension control in a diverse general cardiology practice. J Clin Hypertens (Greenwich).

[20] Heisler M, Piette JD, Spencer M, Kieffer E, Vijan S (2005). The relationship between knowledge of recent HbA1c values and diabetes care understanding and self-management. Diabetes Care.

[21] Stewart SF, Moon Z, Horne R (2023). Medication nonadherence: health impact, prevalence, correlates and interventions. Psychol Health.

[22] Ding A, Dixon SW, Ferries EA, Shrank WH (2022). The role of integrated medical and prescription drug plans in addressing racial and ethnic disparities in medication adherence. J Manag Care Spec Pharm.

[23] Aremu TO, Oluwole OE, Adeyinka KO, Schommer JC (2022). Medication adherence and compliance: recipe for improving patient outcomes. Pharmacy (Basel).

[24] Lee S, Godwin OP, Kim K, Lee E (2015). Predictive factors of patient satisfaction with pharmacy services in South Korea: a cross-sectional study of national level data. PLoS One.

[25] Mularczyk-Tomczewska P, Gujski M, Koperdowska JM, Wójcik J, Silczuk A (2025). Factors influencing patient satisfaction with healthcare services in Poland. Med Sci Monit.

